# The Role of Adherence and Retreatment in *De Novo* Emergence of MDR-TB

**DOI:** 10.1371/journal.pcbi.1004749

**Published:** 2016-03-11

**Authors:** Dominique Cadosch, Pia Abel zur Wiesch, Roger Kouyos, Sebastian Bonhoeffer

**Affiliations:** 1 Institute for Integrative Biology, ETH Zurich, Switzerland; 2 Division of Epidemiology of Microbial Diseases, Yale School of Public Health, New Haven, Connecticut, United States of America; 3 Department of Pharmacy, Faculty of Health Sciences, Norwegian Arctic University (UiT), Tromsø, Norway; 4 Division of Infectious Diseases and Hospital Epidemiology, University Hospital Zurich, University of Zurich, Switzerland & Institute of Medical Virology, Swiss National Center for Retroviruses, University of Zurich, Zurich, Switzerland; Emory University, UNITED STATES

## Abstract

Treatment failure after therapy of pulmonary tuberculosis (TB) infections is an important challenge, especially when it coincides with *de novo* emergence of multi-drug-resistant TB (MDR-TB). We seek to explore possible causes why MDR-TB has been found to occur much more often in patients with a history of previous treatment. We develop a mathematical model of the replication of *Mycobacterium tuberculosis* within a patient reflecting the compartments of macrophages, granulomas, and open cavities as well as parameterizing the effects of drugs on the pathogen dynamics in these compartments. We use this model to study the influence of patient adherence to therapy and of common retreatment regimens on treatment outcome. As expected, the simulations show that treatment success increases with increasing adherence. However, treatment occasionally fails even under perfect adherence due to interpatient variability in pharmacological parameters. The risk of generating MDR *de novo* is highest between 40% and 80% adherence. Importantly, our simulations highlight the double-edged effect of retreatment: On the one hand, the recommended retreatment regimen increases the overall success rate compared to re-treating with the initial regimen. On the other hand, it increases the probability to accumulate more resistant genotypes. We conclude that treatment adherence is a key factor for a positive outcome, and that screening for resistant strains is advisable after treatment failure or relapse.

## Author Summary

Our ability to treat and control acute pulmonary tuberculosis (TB) is threatened by the increasing occurrence of multi-drug-resistant tuberculosis (MDR-TB) in many countries around the globe. It is not clear whether MDR-TB occurs predominantly due to transmission, or whether there is a substantial contribution due to *de novo* emergence during treatment. Understanding the underlying mechanisms that are involved in the emergence of MDR-TB is important to develop countermeasures. We use a computational model of within-host TB infection and its subsequent treatment to qualitatively assess the risks of treatment failure and resistance emergence under various standard therapy regimes. The results show that especially patients with a history of previous TB treatment are at risk of developing MDR-TB. We conclude that *de novo* emergence of MDR-TB is a considerable risk during treatment. Based on our findings, we strongly recommend widespread implementation of drug sensitivity tests prior to the initiation of TB treatment.

Sebastian Bonhoeffer is a Deputy-Editor-in-Chief for *PLOS Computational Biology*.

## Introduction

Tuberculosis (TB) is a key challenge for global health [1,2]. At present about one third of the global population is latently infected [3] and every year about 1.7 million people die of tuberculosis. A large number of patients live in resource-limited settings with restricted access to health-care. It is imperative that standard treatment measures are assessed for their efficacy and reliability.

Understanding the driving forces behind therapy failures is challenging. This is to a large extent the case because of the complex life cycle and population structure of TB: The typical sequence of events leading to acute pulmonary tuberculosis occurs as follows [1,4–7]. Upon inhalation, TB bacilli reach the pulmonary alveoli of the lung. There they are assimilated by phagocytic macrophages. In most cases the bacteria are being killed continuously by phagocytosis while the cell-mediated immunity develops. More rarely, they may persist in an inactive state, which is considered a latent infection. Infected macrophages may aggregate and form granulomas by recruiting more macrophages and other cell types. Inside granulomas, increased necrosis of macrophages can lead to the formation of a caseous core. In latently infected hosts, an equilibrium establishes where the immune system prevents further growth but the bacteria persist in a dormant state [8,9]. However, especially in patients with a compromised immune system, the bacteria may continue or resume growth [4,6]. In this case, the bacterial population steadily increases until the granuloma bursts into the bronchus forming an open cavity. Mycobacterium tuberculosis is an aerobic organism and depends on the availability of oxygen to promote its growth. Because the oxygen levels inside macrophages and granulomas are low, the growth rate is reduced [6,9–13]. In open cavities, oxygen supply is not limiting anymore and the population size increases rapidly. The extracellular bacteria in the cavities may also spread to other locations in the lung where they are again combated by the dendritic cells of the immune system. Some bacteria can be expelled with sputum and be transmitted to other individuals or they may enter a blood vessel and cause lesions in other organs.

The standard treatment is a six-month short-course regimen [1,14–17], consisting of two months of combination therapy with isoniazid, rifampicin, pyrazinamide and ethambutol followed by a continuation phase of four months with isoniazid and rifampicin only [18]. According to tuberculosis treatment guidelines all drugs are taken daily during the first two months. During the following four months isoniazid and rifampicin are administered three times a week with a 3-fold increased isoniazid dose [15]. For patients with previous TB treatments the WHO recommends a 8-month retreatment regimen containing additionally streptomycin [17].

**Fig 1:**
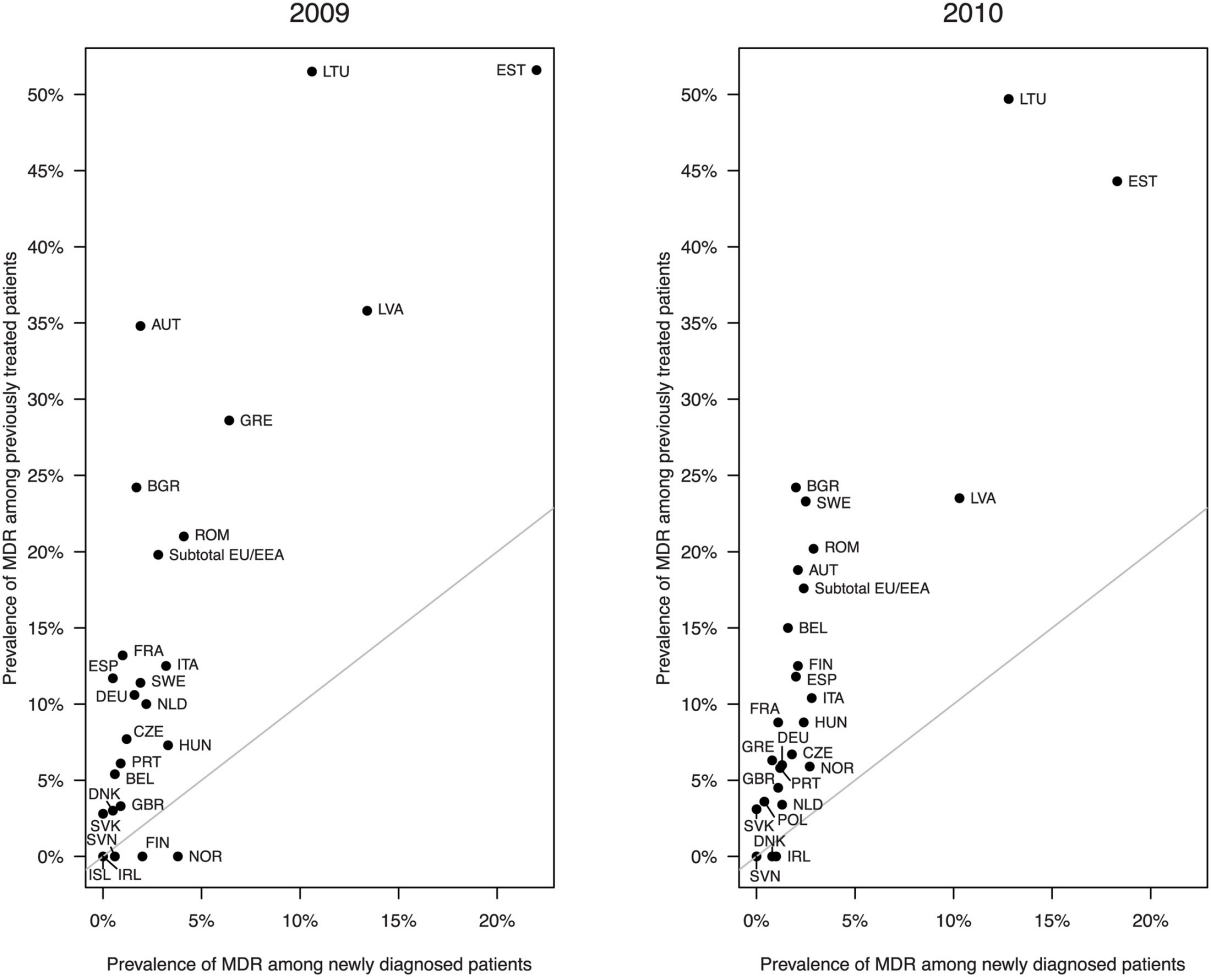
The prevalence of multidrug-resistant tuberculosis (MDR-TB) in most European countries is higher among previously treated patients than among newly diagnosed patients. The data on the percentage of newly diagnosed and previously treated patients with MDR-TB where taken from reference [35] for 2009 and from reference [36] for 2010. Countries with incomplete data were omitted.

In recent years, the problem of drug resistance has increased in severity due to the emergence and spread of multi-drug-resistant tuberculosis (MDR-TB) [19–21], where MDR-TB is defined as infection by *M*. *tuberculosis* strains conferring resistance to at least isoniazid and rifampicin. Resistant TB is assumed to emerge at least in part due to inappropriate treatment or suboptimal adherence to the treatment regimen [22]. Poor compliance has been associated with treatment failure and the emergence of resistance in previous studies [23–27]. Multi-drug-resistance usually develops in a step-wise manner. These steps are thought to include functional monotherapy; either due to different drug efficacies among certain bacterial populations or due to different pharmacokinetics [28,29]. Prevalence data of MDR-TB in Europe (see Fig 1) show that patients who have previously received treatment are on average six times more likely to suffer from MDR-TB than patients who are newly diagnosed. There are several possible explanations for this observation. Individuals who are infected with MDR-TB are more likely to have a treatment failure or a later relapse [30–33], especially if they are not properly diagnosed. These patients could then come under more accurate scrutiny and eventually be reported as MDR-TB patients with previous treatment history. Another more direct possibility is that a considerable fraction of patients who have contracted susceptible TB develop *de novo* MDR-TB during the first therapy [34].

The goal of this study is to assess the factors that determine the *de novo* acquisition of drug resistance and to get a better insight in the underlying dynamics. Specifically, we want to study the contribution of imperfect compliance and retreatment regimens. In some areas, second-line drugs are not easily accessible. Moreover, drug-susceptibility tests may not be performed due to the lack of required infrastructure or questionable reliability of patient treatment history [37]. Hence, we assess the impact of a retreatment that is identical to the first therapy as well as a retreatment that follows the WHO recommendation [17]. To achieve this goal we develop a computational model of a within-host TB infection and its consecutive treatment with currently recommended first-line regimens. The model framework encompasses the population dynamics of various *M*. *tuberculosis* genotypes with different resistance patterns in three pulmonary compartments as well as the pharmacodynamics and the pharmacokinetics of the drugs that are used for treatment. The aim is to provide qualitative insights into the infection dynamics of tuberculosis. The parameterization is based on the most recent concepts and individual experimental results found in the literature. Given the current lack of a good animal or *in vitro* model for TB, a computational model, may help to bridge the gaps arising from the inaccessibility of TB in experimental model systems and allow the hypothetical assessment of treatment scenarios, which would be otherwise ethically inadmissible in patient trials. In particular, problems resulting from imperfect therapy adherence can be usefully addressed with a computational model.

## Methods

**Fig 2:**
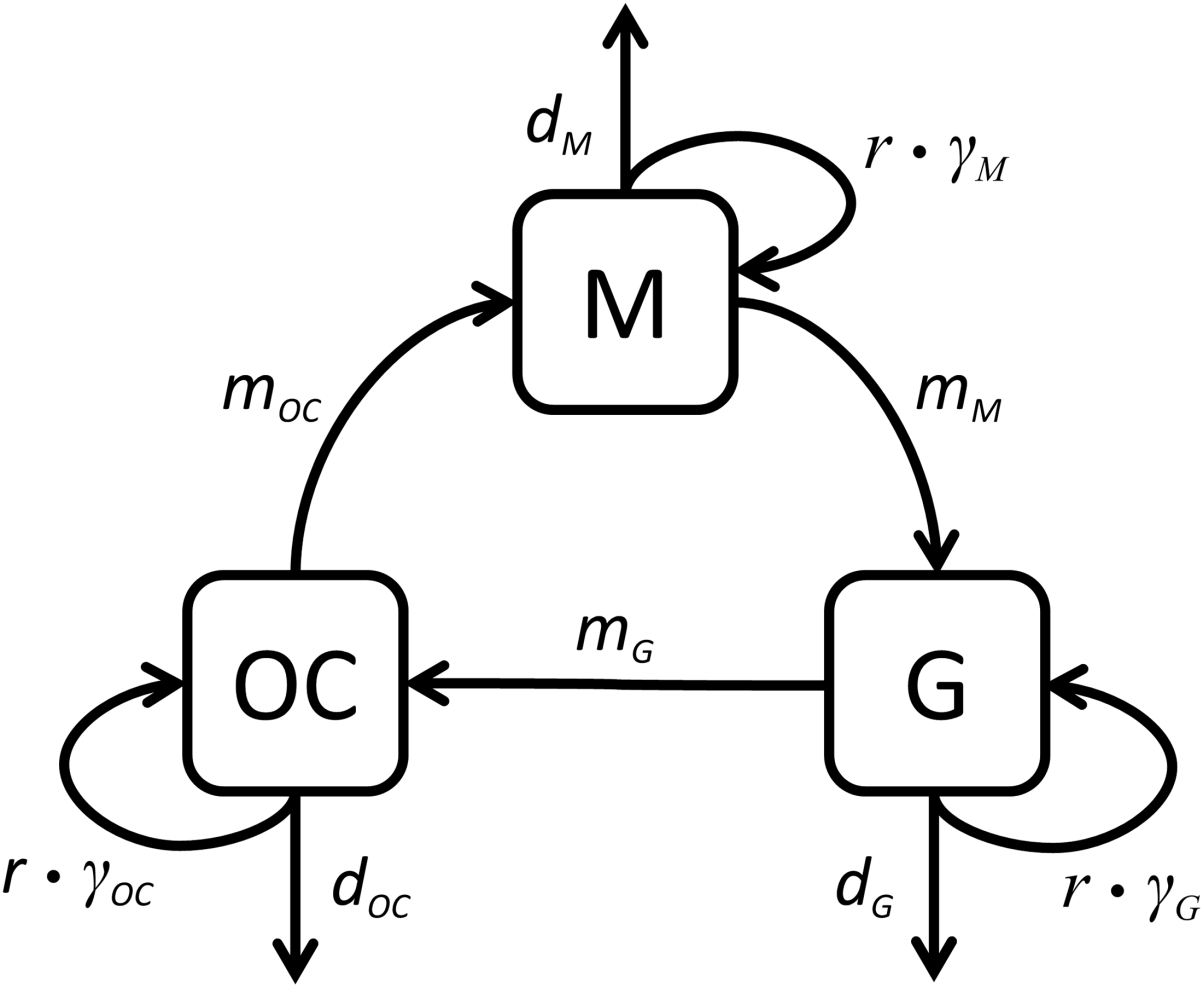
Diagram of model for the pathogenesis during acute pulmonary tuberculosis infection. We consider three different physiological compartments for the location of TB bacteria: host macrophages (M), granulomas (G) and open cavities (OC). The base replication rate *r* of the bacteria is modified by a compartment specific parameter γ. The bacteria die with a density-dependent rate *d* and migrate from one compartment to another at a rate *m*.

In the following section we present the basic framework of the computational model, the parameterization and key aspects of our simulations. In essence, our model consists of coupled logistic-growth models that are connected such that they capture the basic population structure (compartments) of TB (see Fig 2). The action of TB-drugs is included in this model via realistic pharmacokinetics / pharmacodynamics functions. Resistance to these drugs is modeled by distinguishing between up to 32 genotypes (all combinations of 5 mutations) with varying resistance patterns. Since mutations are generated at low frequencies and numbers (due to the low bacterial mutation rate), chance events are essential in the dynamics of this system and hence we consider a stochastic version of the model. In the following we provide a detailed description of the model; the model equations and further details can be found in S1 Text.

### Model

Our model describes pulmonary tuberculosis and assesses the emergence of resistance during multi-drug therapy. A graphical illustration of the model is provided in Fig 2. The model reflects the compartmentalization of the bacteria into three distinct subpopulations as described by Grosset [5]: intracellular bacteria within macrophages (M), bacteria within the caseating tissue of granulomas (G) and extracellular bacteria which mostly reside in open cavities (OC). The compartments differ in their maximum population sizes as well as the bacterial replication rates that they allow. The base replication rate *r* is modified by a factor *γ*, which reflects the compartment specific conditions that influence the replication rate. Bacteria have a natural density-dependent death rate in each compartment. The constant replication rate and the density-dependent death rate constitute a logistic growth model that was assumed to describe the basic population dynamics. Bacteria also migrate unidirectionally at a rate *m* from one compartment to another. Offspring bacteria have a certain chance to acquire or lose a mutation that confers resistance to one out of up to five drugs that may be administered during treatment. Every resistance mutation confers a fitness cost which affects the reproductive success of its carrier. This means that the bacterial population inside a compartment comprises of up to 32 genotypes, which differ in their drug resistance pattern as well as their relative fitness.

To outline the population dynamics within a single compartment we describe them first in the form of a deterministic differential equation. The dynamical equation is given by

$$\frac{dN_{c,g}}{dt}=r\cdot \gamma_{c}\cdot \omega_{g}\cdot N_{c,g}-m_{c}\cdot \frac{N_{c}}{K_{c}}\cdot N_{c,g}+m_{c\prime }\cdot \frac{N_{c\prime }}{K_{c\prime }}\cdot N_{c\prime ,g}-(d_{c}+\kappa_{c,g})\cdot N_{c,g}$$ (1)

Here *N*_*c*,*g*_ is the number of bacteria of a specific genotype *g* in a specific compartment *c*. The parameter *r* is the base replication rate of *M*. *tuberculosis* and ***γ***_*c*_ is a factor, which modifies the replication rate according to the different metabolic activities in each compartment. *ω*_*g*_ represents the relative fitness of the specific genotype. *m*_*c*_ is the rate with which bacteria migrate to the subsequent compartment. The migration rate is multiplied by the ratio between the total population size *N*_*c*_ and the carrying capacity *K*_*c*_. This reflects the increased migratory activity that takes place during an acute infection. *N*_*c´*_, *K*_*c’*_ and *m*_*c´*_ correspond to the overall bacterial population including all genotypes of the supplying compartment, its carrying capacity and its migration rate, respectively. The last term reflects the density-dependent death rate *d*_*c*_ and the drug induced genotype specific killing rate *κ*_*c*,*g*_. The bactericidal effects of the drugs contribute additively to the killing rate *κ*_*c*,*g*_ (see S1 Text for further details).

The dynamics of the bacterial population in the model are actually simulated as stochastic processes. For this reason we translated the underlying deterministic differential equations into a corresponding stochastic framework by applying Gillespie’s τ-leap method [38].

### Parameterization

The parameter estimates used in this model are whenever possible drawn or derived from experimental results in the literature. To account for the diversity of infection and treatment courses in different patients we allow some parameters to vary within a certain range. Parameters are summarized in Table 1.

**Table 1:** Compartment characteristics.

	Macrophages	Granulomas	Open cavities
*Compartmental characteristics*			
Carrying capacity (*K*_*c*_)	10^5^–10^7^ [5]	10^5^–10^7^ [5,83]	10^8^–10^10^ [4,5,39,83]
Growth modifier (*γ*_*c*_)	0.5 [84,85]	0.1 [5]	1
Migration rate (*m*_*c*_, *m´*, *d*^*-1*^)	0–0.1^a^	0–0.1^a^	0–0.1^a^
*Relative drug efficacies*			
Isoniazid	0–1 [57,59,68]	0.01 [69]	1
Rifampicin	0.01 [59]	0.01 [6]	1
Pyrazinamide	0 [58,66]	1 [6]	0 [67]
Ethambutol	1 [59]	0–1 [6,65,70]	1
Streptomycin	0.1 [56,57]	0.01 [6]	1

The provided references support the order of magnitude of the parameters, not the exact value.

^a^ estimation

The basic growth dynamics rest upon the replication rate and the carrying capacity of the compartments. Based on recent studies [39–41] we assume a maximum bacterial load between 10^5^ and 10^7^ bacteria each for the macrophage and the granuloma compartment and 10^8^ to 10^10^ bacteria for the extracellular compartment. Under optimal conditions *M*. *tuberculosis* has a replication time of 20*h*, hence we set the maximum replication rate in the model to 0.8 *d*^-1^ [5].

Every new bacteria cell has at birth the chance to acquire or lose one or multiple resistance mutations and therefore get a genotype, which is different from the mother cell. The frequency of specific resistance mutations and therefore the mutation rate for the main first-line drugs have been first estimated by David in 1970 [42] to be around 10^−7^–10^−10^. However, more recent observations suggest considerably higher frequencies in the order of 10^−6^ to 10^−8^ [5,43]. A possible reason for this discrepancy between these estimates are varying mutation rates in *in vitro* experiments compared to the conditions encountered *in vivo* due to stress-induced mutagenesis mechanisms or variations among strains [44–46]. Furthermore, we assume that mutations only occur during proliferation while mutations during the stationary phase could serve as an additional source of resistance mutations [47]. Therefore, we choose to allow for patients with the more recent higher mutation rates because this will yield more conservative estimates (see Table 2). Our model incorporates backwards mutations from the resistant to the sensitive phenotype, which also restore the reproductive fitness. However, we consider a reversion to be ten times less likely than the original forward mutation because the occurrence of any additional mutation within a gene to be an exact reversion is more infrequent.

**Table 2:** Model parameters.

	Isoniazid	Rifampicin	Pyrazinamide	Ethambutol	Streptomycin
Half-Life (*h*^*-1*^)	FA^a^: 1.54 ± 0.30 [86]	2.46 [86]	9.6 ± 1.8 [86]	2.6 [87]	3 [88,89]
	SA^a^: 3.68 ± 0.59 [86]				
Dose (*mg/L*)	FA^a^: 2.80 ± 0.71^b^ [89]	13.61 ± 3.96 [89]	29.21 ± 4.35 [89]	5.0 [87]	35–45 [14,89]
	SA^a^: 4.26 ± 0.94 ^b^ [89]				
*MIC (mg/L)*	0.025 [57]	0.4 [57]	28 [58]	1.0 [57]	0.5 [54,56]
*EC*_*50*_ *(mg/L)*	0.033 ^d^	0.51 ^d^	40 ^c^	0.20 ^d^	0.32 ^d^
*E*_*max*_	1.86 ^d^	1.82 ^d^	1.94 ^d^	0.96 ^d^	1.31 ^d^
*C*_*ELF*_*/C*_*Serum*_ (*ρ*) ^e^	FA^a^: 1.74–5.88 [90]	0.34 [90]	13.60–24.76 [90]	0.92–1.13 [90]	1 ^c^
	SA^a^: 1.37–5.69 [90]				
Resistance frequency	2.56 · 10^−8^–10^−7^ [42,43]	2.25 · 10^−10^–10^−8^ [42,43]	10^−9^–10^−8^ ^c^	10^−7^ [42]	2.95 · 10^−8^–10^−7^ [42],^c^
Resistance cost	0.1 [49]	0.1 [50,51]	0.1 [49]	0.1 ^c^	0.1 [49,50]

Some of the provided references support the order of magnitude of the parameters, not the exact value.

^a^ FA = fast acetylators, SA = slow acetylators

^b^ If isoniazid is administered three times a week instead of daily the dosage is three times higher [14,91]

^c^ estimation

^d^ see text

^e^ ELF = epithelial lining fluid

When assessing the prevalence of certain genotypes, fitness costs that come with resistance mutations have to be considered. The cost of resistance against anti-tuberculosis drugs appears generally to be low [48–51]. Drug-resistant mutants isolated in patients have even been found to be on par with susceptible wild type strains regarding their infectivity and replicative potential. Since cost-free resistance mutations are rather rare, the high fitness of resistant strains that have been found in clinical isolates [48,49] is assumed to arise due to the acquisition of secondary site mutations which minimize the fitness costs (so-called compensatory mutations) [50]. However, there is evidence that at least initially newly acquired drug resistance confers some physiological cost [52]. Because our model simulates the *de novo* acquisition of resistance mutations and because the time frame of a single patient treatment is rather short we assign a small fitness cost to every possible mutation and neglect the counterbalance of fitness costs by compensatory mutations.

The effect of administered drugs depends on the pharmacokinetics and pharmacodynamics of these drugs (see Table 1). Both influence the killing rate *κ*_*c*,*g*_ at any given time point during treatment. While pharmacokinetic parameters describe the course of the drug concentration in the target tissue, pharmacodynamic parameters characterize the effect the drugs have at a given concentration. The minimal inhibitory concentration (*MIC*) describes the minimal drug concentration at which bacterial growth is reduced by at least 99%. Additionally, the *EC*_*50*_ describes at which drug concentration the half-maximal effect (commonly, bacterial killing) is observed, while the *E*_*max*_ indicates the maximal effect of the drug. These pharmacodynamic parameters are obtained by fitting the drug action model to killing curves found in the literature [53,54] (see S1 Text). The specific efficacy of most drugs in the different compartments is typically not quantified. There are several studies that tried to assess the bactericidal activity inside macrophages [55–59]. Unfortunately, these estimates are highly variable and sometimes even contradictory [55,58]. In addition to these experimental difficulties, it is possible that the pharmacodynamics of anti-tuberculosis drugs are again different in the human body [60–64]. To reflect this uncertainty we assign compartment efficacies from a range of values which corresponds to the most recent estimates [56–59,65–70].

### Simulations

To investigate the role of treatment adherence on patient outcome, we followed disease progression starting with the infection of macrophages until all compartments approximately reached their maximum bacterial load. For each parameter set, we simulate the outcome of 10’000 patients who vary both in their pharmacokinetic and–dynamic characteristics as well as compartmental attributes. Parameters are generally picked from a normal distribution. If only a range is known the parameters are chosen from a uniform distribution. To measure the actual treatment efficacy we let every patient develop an acute tuberculosis infection during 360 days. This allows for the emergence of mutants prior to treatment initiation and provides enough time for the establishment of an equilibrium in the bacterial population composition. After this period we start the standard short course therapy regimen with four drugs being taken daily for two months followed by four months in which just isoniazid and rifampicin are taken three times per week. If the infection is not completely sterilized after the first treatment we schedule a retreatment. Since the model does not cover the possibility of dormant bacteria the population recovers rather quickly after an unsuccessful treatment. Hence, we begin the retreatment 30 days after completion of the previous treatment. After such a time span the population reaches a bacterial load where acute symptoms would be again suspected. If not stated otherwise the retreatment corresponds to the WHO recommendation for retreatments [31,71]. The WHO recommendations include streptomycin, which is used together with the original four first-line drugs during the first two months. Afterwards the therapy is being continued for another month without streptomycin and during the last five months only isoniazid, rifampicin and ethambutol are administered. All drugs are being taken daily during the whole retreatment.

The 95% confidence intervals (CI) of patient outcomes in the figures is calculated by picking the value for a two-sided 95% confidence limit with *n*– 1 degrees of freedom from a *t*-distribution table where *n* is the number of patients. This value is then multiplied with the standard deviation *σ* and divided by the square root of *n*. The resulting value is then added and subtracted from the mean to get the actual confidence interval.

$$CI=\frac{t_{n-1}^{95\%}\cdot \sigma }{\sqrt{n}}$$ (2)

## Results

### Treatment efficacy in single compartments against wild-type TB and MDR-TB

**Fig 3:**
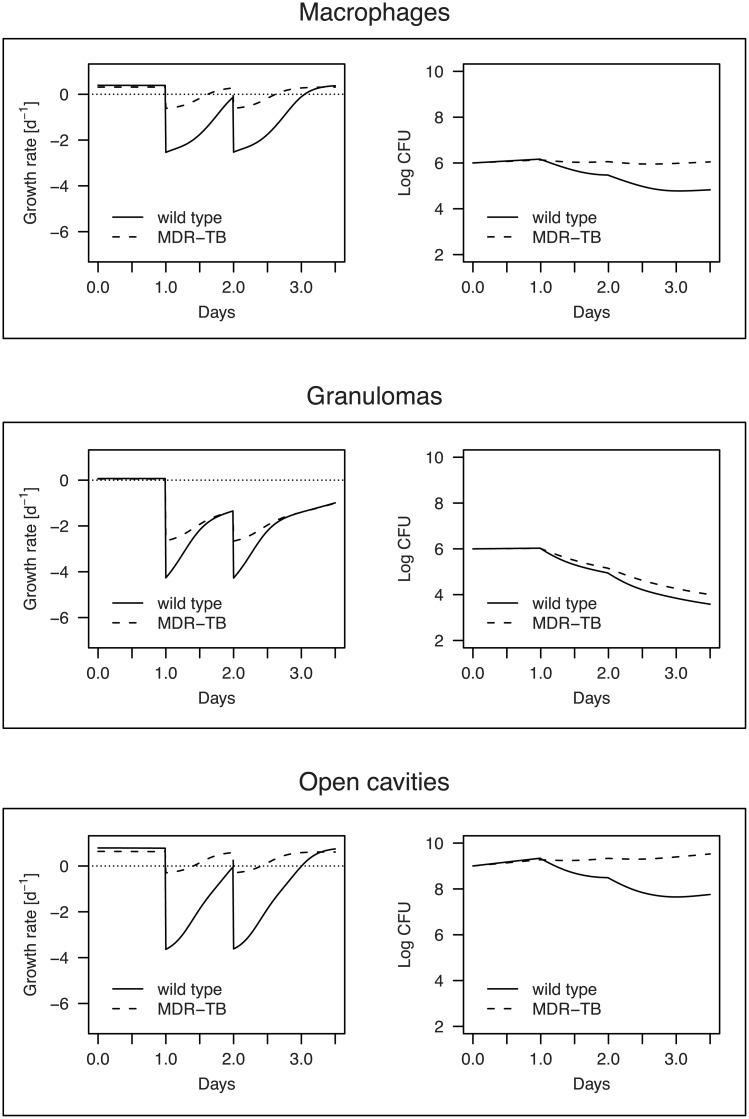
Net growth rates and population dynamics of wild-type and MDR bacteria in the modeled compartments after two days of treatment with the four first line drugs. All parameters for which a range of values exist have been set to the median value. On day 1 and day 2 all four drugs are applied simultaneously.

The impact of treatment on the net growth rate of wild-type or MDR bacteria differs strongly between compartments (Fig 3): Before treatment starts, the growth rates in macrophages and granulomas are lower than in the open lung cavities due to hypoxia and a generally adverse environment for bacterial growth in these compartments. Since we assume that the drug concentration immediately reaches the maximum the impact of combination therapy on growth rate is immediately apparent after the administration of the first dose of drugs. In all compartments the drugs are able to keep the wild-type populations from regrowth during the following days. Especially in granulomas pyrazinamide is able to diminish the population over a long period due to its relatively long half-life. MDR-TB is substantially less affected by the combination therapy because only pyrazinamide and ethambutol are effective. This means that in macrophages or open lung cavities the multi-drug-resistant population remains constant at best or is even able to slowly grow. Only in the granulomas where mostly pyrazinamide is active (see Table 1) the loss of effectiveness of isoniazid and rifampicin is less prominent.

### The role of adherence

The compliance of a patient with the prescribed drug regimen is a key factor for a successful treatment outcome. For the assessment of treatment success we monitor for every patient three different nested treatment outcomes. Firstly, we define treatment failure as the incomplete sterilization of the lung at the end of the therapy. Secondly, the emergence of MDR-TB is defined in our simulations as 10% or more [72] of the remaining bacterial population after treatment failure being resistant against at least isoniazid and rifampicin. Finally, emergence of full resistance (FR) is defined as 10% or more of the population being resistant against all drugs that were used in the treatment regimen (either 4 drugs for first treatment or up to 5 drugs for retreatment).

Adherence in our simulations refers to the probability with which the patient takes the prescribed drugs at any given day. We assume that failure to take drugs on a given day always affects all drugs of the prescribed regimen.

**Fig 4:**
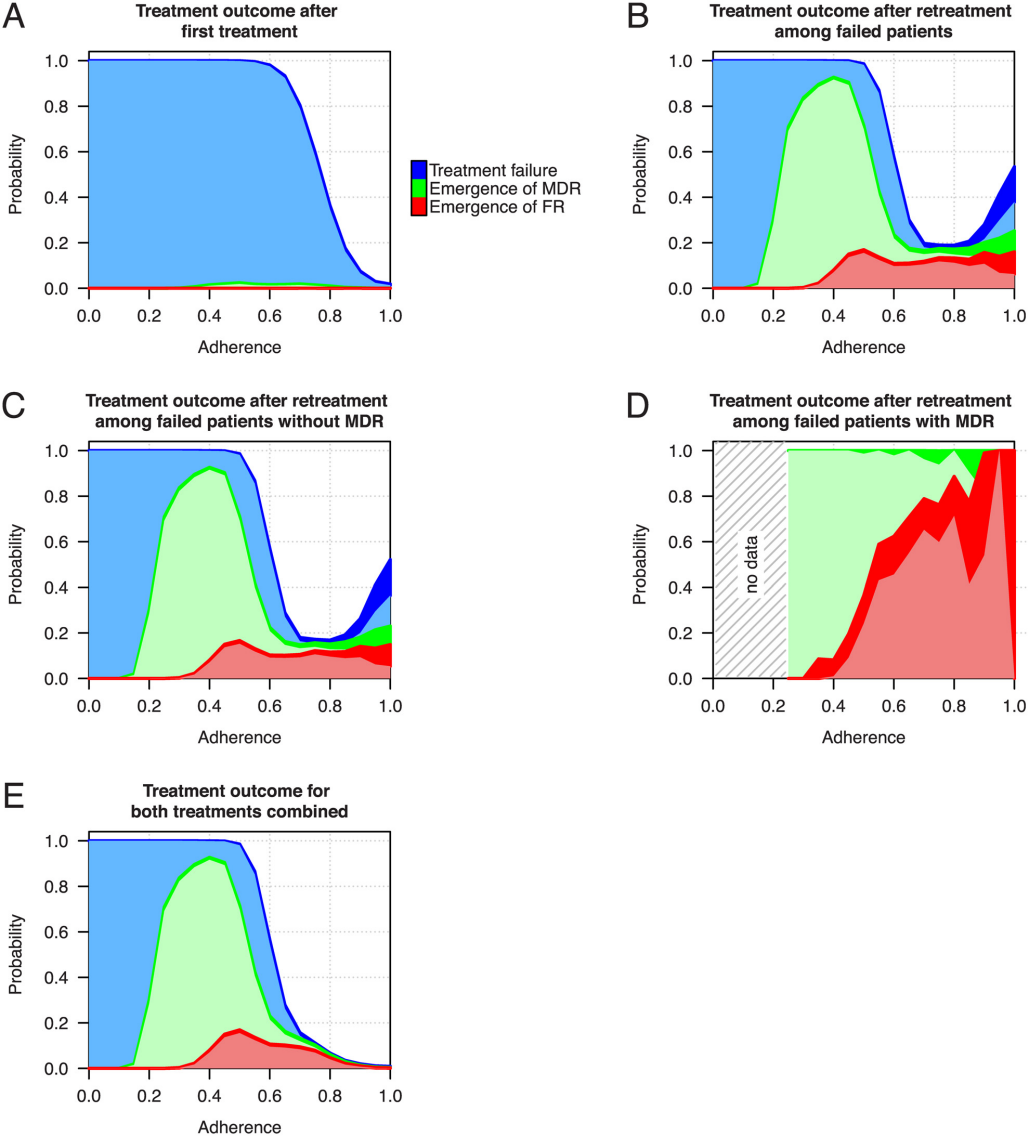
Probabilities for treatment failure (blue), emergence of MDR-TB (green) and the emergence of a fully resistant strain (FR, red). (A) Treatment outcome probabilities based on the assessment of 10,000 simulated patients undergoing six month short course therapy at different levels of adherence. (B) Outcome probabilities of the standard retreatment regimen containing streptomycin for patients failing the previous treatment. (C and D) Retreatment outcome probabilities for patients failing the first treatment without or with MDR-TB respectively. (E) The overall probabilities for treatment outcome when both treatment regimens are considered. The width of the dark colored areas indicate the 95% confidence interval. Please note that the colored areas overlap and share a common baseline. Therefore, FR is a subcategory of MDR and FR and MDR are subcategories of treatment failure. The confidence intervals for the retreatment tend to widen at higher adherence levels due to the lower number of patients failing the previous treatment. The area with no data in panel (D) arises because patients with low adherence do not harbor MDR-TB after the first treatment.

In our simulations, the level of adherence has a strong but complex impact on treatment success (Fig 4A). Under perfect adherence the model shows a very low failure rate. However, if adherence decreases the probability for treatment failure increases rapidly. Between 40% and 80% adherence there is also a small fraction of patients that fail treatment due to the emergence of MDR-TB. Furthermore, at these adherence levels the model also shows only limited treatment success. Thus, failure decreases monotonically with adherence while MDR is maximized at intermediate levels. Patients who fail on the first treatment and who undergo retreatment (Fig 4B) have a failure rate of 20% at 80% adherence. However, the probability for treatment failure increases to about 50% under perfect adherence. Patients who fail the first treatment despite high adherence may often have disadvantageous combinations of PK/PD parameters, which also decrease their success probabilities during the retreatment. In Fig 4B, 4C and 4D the number of patients per adherence level undergoing retreatment decreases strongly as can be seen from the frequency of treatment failure in Fig 4A. When comparing Fig 4A and 4E, which shows the combined outcome probabilities for both treatments, we see that the retreatment reduces the probability of failure over the upper half of the adherence spectrum.

### The role of retreatment

The additional treatment success of retreatment regimens depends on adherence and the addition of streptomycin to the regimen (Fig 4B). In our model, even under perfect adherence the chance of treatment failure remains substantial, and in the majority of patients who fail under retreatment MDR-TB emerged *de novo*. Furthermore, at suboptimal adherence levels a considerable proportion of patients even carry strains that are not susceptible to any of the five administered drugs. The outcome of retreatment depends crucially on whether MDR was acquired during initial treatment: Because the majority of patients who fail the first treatment do not carry MDR-TB their outcome probabilities for the retreatment are almost identical to the overall cohort of failed patients (Fig 4C). Even though the vast majority of patients who failed the first treatment did not develop MDR-TB, a substantial fraction of patients who also failed the second treatment harbor MDR or FR strains. This occurs due to increased subpopulations of monoresistant bacteria that accumulate during the first treatment and that are by itself not sufficient to be diagnosed as MDR-TB. When comparing patients who are diagnosed with MDR-TB after the first treatment (Fig 4D) and those who are not (Fig 4C) we see that patients who develop MDR-TB are very likely to fail the retreatment as well. At higher adherence levels the majority of those patients develops full resistance against all five drugs (Fig 4D). When considering the outcome for both treatments combined (Fig 4E) it becomes more evident that the addition of streptomycin and the more intense retreatment has a beneficial effect on the overall success rate but patients who also fail the retreatment are more likely to carry multidrug-resistant TB strains.

**Fig 5:**
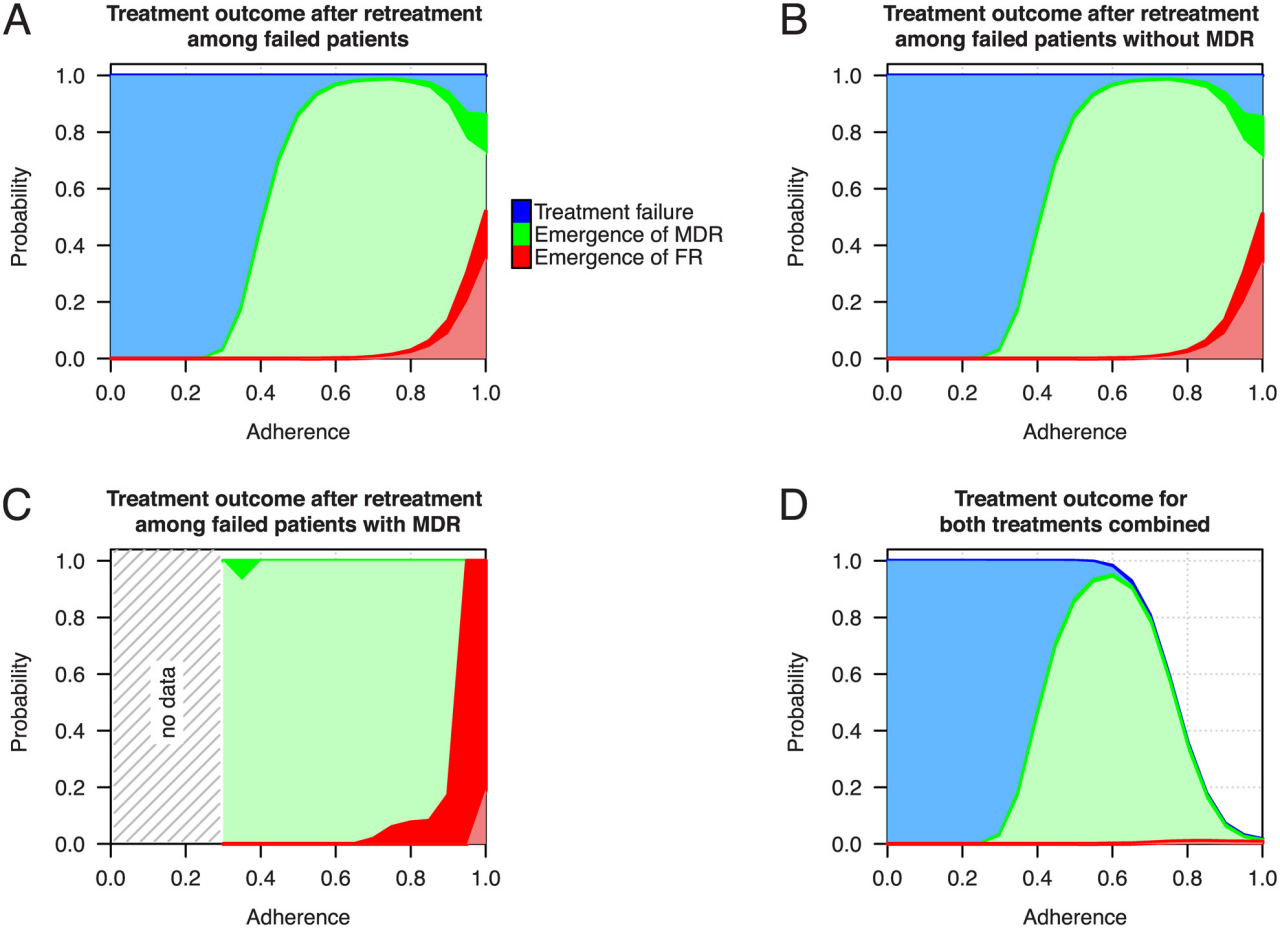
Corresponding treatment outcomes after two rounds of identical six-month short course therapy. (A) Treatment outcome probabilities after two rounds of identical first line therapy for treatment failure (blue), the emergence of MDR-TB (green) and the emergence of a fully resistant strain (FR, red). (B) Probabilities for patients who did not complete the first therapy successfully but who also did not harbor MDR-TB. (C) Treatment outcome probabilities for patients who failed the first treatment with MDR-TB. (D) The overall probabilities for treatment outcome when both treatment regimens are considered. There is no data available in panel (C) for patients with a lower adherence than 25% because such patients did not harbor MDR-TB after the first treatment.

When second-line drugs are not available or susceptibility test are not performed, it may occur frequently that a previously treated patient is retreated with the first line treatment. Our results in Fig 5 show that such a retreatment with the first line drugs has almost no additional treatment success beyond the initial treatment. Patients all across the spectrum of adherence experience treatment failure. The identical first-line retreament only increases the chances for the bacteria to accumulate resistance mutations and leads between 50% to 100% adherence to nearly all uncleared patients harboring MDR-TB or worse. This outcome is standing out when comparing the cumulative treatment success in Fig 5D with the results after the first treatment. While the overall success curve did not change the fraction of MDR-TB patients over a large adherence range increased substantially.

## Discussion

The aim of this study is to elucidate the effects of treatment adherence and retreatment on the emergence of resistance in TB. The model explicitly incorporates the pharmacodynamics and pharmacokinetics of all drugs that are used for standard therapy and the WHO retreatment recommendation. Depending on the compartment in the lung in which the bacteria reside (macrophages, caseous centers of granulomas or open cavities), *M*. *tuberculosis* has different stages of infection and drug-susceptibilities. Therefore, we explicitly include these different compartments to be able capture the effect of heterogeneous selection pressure. Because not all of the parameters used in our model have been quantified with high accuracy, we do not claim that the model has quantitative predictive power. Rather, it aims to qualitatively demonstrate the underlying dynamics of a tuberculosis infection.

Our results suggest that poor adherence is a major cause for treatment failure. When considering the predicted rates of treatment failure one also has to take into account that our definition of treatment failure is probably rather conservative. We do not include the possibility of remaining dormant bacteria, which might increase the likelihood of treatment failure or relapse. On the other hand, we also neglect the possibility of the infection being contained at a later time point by the immune system, thus probably underestimating the chance of success. It is also noteworthy that even at perfect adherence some patients may have a negative treatment outcome. This is most likely due to a random aggregation of very adverse pharmacokinetic parameters and unfavorable infection attributes in some patients. Such outcomes due to pharmacokinetic variability and despite good adherence have been predicted in an *in vitro* study [73]. Furthermore, our results show that over a certain range of adherence a small fraction of patients develop MDR-TB. At intermediate adherence these patients also have a low likelihood of being treated successfully. Thus, good adherence to therapy is crucial: Not only does it increase treatment success, it also decreases the probability for the emergence of MDR-TB.

According to our model, the WHO recommendation for retreatment is somewhat of a double-edged sword. While at high adherence levels the recommended treatment is able to cure the majority of patients who failed the first line therapy, it also increases the fraction of patients harboring drug resistant strains across almost the whole spectrum of adherence. Previous studies already raised concerns about the possible amplification of resistance [71,74–77]. In the WHO treatment guidelines it is recommended that drug susceptibility test results should be taken into account when deciding upon the retreatment regimen [17]. However, the vast majority of patients in our model would probably not have been diagnosed with MDR-TB after the first regimen even though they may still harbor increased subpopulations of monoresistant bacteria. Therefore it is conceivable that many would have been treated with the WHO recommended regimen. A large fraction of patients who failed this retreatment eventually developed MDR-TB. Considering the results from our model further clinical studies are needed which analyze the treatment success rates and the accompanying risks of the standard retreatment regimen.

Retreating failed patients with an identical short course therapy leads to poor outcome in our simulations. A lower success rate for MDR-TB patients treated with the standard short-course therapy has been confirmed in a large cohort study [37]. In our simulations it is rare that patients who failed the previous treatment are cured after undergoing the same therapy again provided that adherence remains unchanged. Retreatment with the same regimen only generates more opportunities for single resistant mutants that emerged during the first treatment to accumulate further mutations, thus minimizing the number of future treatment options.

These findings are in accordance with previous studies which found a positive correlation between previous treatment and the occurrence of resistance [78–81]. This might be an indicator that *de novo* resistance on an epidemiological scale occurs at a significant frequency and that the main contributor to the frequency of MDR-TB is not necessarily the mere transmission of such strains.

In summary our data show that patient adherence is a crucial component of treatment success. The probably cheapest and most effective way to ensure a positive treatment outcome while also minimizing the risk for the emergence of MDR-TB is to maintain proper patient compliance with the treatment. This supports the Directly Observed Treatment, Short-Course (DOTS) strategy of the WHO, which includes healthcare workers or community health workers who directly monitor patient medication. If treatment fails, thorough tests of drug susceptibility of the remaining infecting population, would be of considerable value. According to our results a retreatment regimen including streptomycin has the potential to increase the overall cure rate, but also increases the fraction of patients who carry drug-resistant strains. A common principle of physicians is to “never add a single drug to a failing regimen” [82] this principle is often not followed in retreatment. A preceding drug sensitivity test might show existing drug resistances and the retreatment regimen could be adapted accordingly. Nonetheless the standard retreatment regimen is still superior to a retreatment with the identical first-line drugs. Such a retreatment is unlikely to achieve a higher overall cure rate and dramatically increases the probability for the emergence of MDR-TB, which reduces further treatment options. This shows that a dependable patient treatment history that is available to the responsible health professional is also important before initiating a treatment regimen.

## Supporting Information

S1 Text Supplementary information. (DOC)
