# Supplementary material for: The Role of Adherence and Retreatment in De Novo Emergence of MDR-TB
Source: PLoS Comput Biol. 2016 Mar 11;12(3):e1004749. doi: 10.1371/journal.pcbi.1004749 (PMC4788301; doi:10.1371/journal.pcbi.1004749)
Supplement: S1 Text — (DOC) [file pcbi.1004749.s001.doc]

**Supporting information**

**Model equations**

The following descriptions contain further details about the model mechanics and specifications of the equations that comprise the mathematical basis in addition to the explanations in the main article (see *Model* in the Methods section).

The model is based on the τ-leap approximation [1]. In our case we do the simulations with a temporal resolution of 10^-2^ *d*. If in a time step a new bacterium is born it mutates and gains or loses resistance to one or several drugs with a likelihood that is equal to the corresponding mutation rate. For every patient and every drug we initially randomly pick a rate from a uniform distribution with the indicated minimum and maximum values in Table 1. The probability for a backward mutation is ten times lower than the corresponding picked rate.

We assume that (uncompensated) resistance alleles confer a fitness disadvantage in the absence of drugs. In our model we restrict the effect of resistance costs *c_l_* to the reduction of the reproductive success. If multiple alleles involve a cost, the fitness of the corresponding genotype is given by

| $w_{g}=\prod_{l=1}^{n} (1-c_{l})$ | ( 3 ) |
| --- | --- |

where *c_l_* is the cost of a resistance allele at the locus *l* and *n* is the number of resistance loci. The susceptible wild-type alleles have no cost and therefore the fully susceptible strain has a fitness of 1.

The death rate *d_c_* of the bacterial population depends on the population density in the compartment among all genotypes.

| $d_{c}=r\cdot\gamma_{c}\frac{N_{c}}{K_{c}}$ | ( 4 ) |
| --- | --- |

where *r* is the maximal replication rate of *M. tuberculosis* and ***γ****_c_* is a factor, which modifies the maximal growth rate according to the different metabolic activities in each compartment. *N_c_* is the combined population size of all genotypes in the compartment and *K_c_* is the carrying capacity of that specific compartment. The higher the population density is, the more increases the death rate. Together with the basic growth function this results in the conventional model for logistic growth:

| $\frac{dN_{c,g}}{dt}=r\cdot N_{c,g}\cdot(1-\frac{N_{c}}{K_{c}})$ | ( 5 ) |
| --- | --- |

The bactericidal activity of the antituberculosis drugs is accounted for by the sigmoid *E_max_* model [2]. This leads to an adapted version of the *enhanced death model* by Czock *et al.* [2] which we extend to reflect the use of multiple distinct drugs . The bactericidal effects of the drugs in a compartment are reflected in the killing rate variable *κ_c,g_*.

| $\kappa_{c,g}=\sum_{d=1}^{n} E_{\max,d}\cdot\left(1-\frac{1}{\frac{C_{d}\cdot\delta_{c,d}\cdot\rho_{d}}{EC_{50,d}}+1}\right)\cdot v_{g,d}$ | ( 6 ) |
| --- | --- |

The killing rate depends on the genotype. Here we assume that a resistant mutant allele confers full resistance to the bactericidal activity of the corresponding drug. The resistance of a given genotype against an antibiotic drug is represented by the boolean variable *v_g,d_* with *v_g,d_* = 0 indicating resistance.

For simplicity we assumed that the drug effects are additive. *n* is the number of drugs and *EC_50,d_* describes the concentration at which the half-maximal kill rate of a specific drug is reached. *E_max_* is the maximal death rate. Together with the *MIC* and the *EC_50_* it determines the antibacterial potency of a drug. *C_d_* is the current drug concentration while *δ_c,d_* and *ρ_d_* are the efficacy of the drug in the specific compartment and the ratio between plasma and epithelial lining fluid concentration, respectively.

We assume that immediately after the uptake of a drug the concentration increases instantaneously by an amount *C_max,d_*., followed by a exponential decay according to the following function,

| $C_{d}(t)=C_{d}(t_{0})\cdot e^{-a(t-t_{0})}$ | ( 7 ) |
| --- | --- |

where *t_0_* is the last time point where the drug has been taken and

| $a=\frac{\ln(2)}{t_{1/2}^{d}}$ | ( 8 ) |
| --- | --- |

where $t_{1/2}^{d}$ is the half-life of drug *d* within the patient.

For simplicity we assume that if the patient is non-adherent on a specific day all due drugs are missed simultaneously. Allowing the drugs to be missed independently caused only a marginally lower chance of a treatment failure (data not shown).

**Fitting of anti-tuberculosis drug action**

The Emax and EC50 values as we use them in our model were not readily available in the literature. In order to obtain them we use again an equation from the *enhanced-death constant-replication model* 76

| $\frac{dN}{dt}=r\cdot N-E_{\max}\cdot\frac{C}{EC_{50}+C}\cdot N$ | ( 9 ) |
| --- | --- |

If we assume that the population has no net growth and therefore the drug concentration *C* is equal to the *MIC* we get the following relation

| $E_{\max}=\frac{r\cdot(MIC+EC_{50})}{MIC}$ | ( 10 ) |
| --- | --- |

The different E_max_ and *EC_50_* values are collected by fitting equation ( 10 ) to the kill curves that were recorded *in vitro* by de Steenwinkel *et al.* and Marcel *et al.* [3,4]. These papers report the *in vitro* effects of constant drug concentrations of isoniazid, rifampicin, ethambutol and streptomycin on the density of a *M. tuberculosis* suspension over the course of six or seven days, respectively. In Figure 6 we show simulations of this experimental setup using our model by assuming a single compartment in which all four available drugs have unimpaired efficacy. The data points were extracted from the original figures as far as the data points were distinguishable. In order to prevent the unpredictable stochastic influence of rescue mutations we remove the possibility of emerging resistance from the model. From the growth curves in the absence of any drug we estimate the average growth rate to be 1.95. This comparably high growth rate is likely due to the adapted phenotypes of regular lab strains of *M. tuberculosis*. The carrying capacity in the assays of de Steenwinkel *et al.* we estimated to be 10^8.5^ and 10^13^ in the assay of Marcel *et al.*

The *MIC* concentrations from the literature [4–6] which are also confirmed in the experimental kill curves serve as reference points at which the bacterial growth and the bactericidal activity of the drug would cancel each other out and the population would stay constant. The EC_50_ concentrations and the interdependent E_max_ values are derived by linear least-square fitting. The best fit values are calculated by averaging over all the available concentrations. The experimental data for isoniazid shows a recovery of population growth after day 2. The authors claim that this effect appears due to the development of a isoniazid-resistant subpopulation [3]. Because we do not consider such rescue mutations we decide to include only the first two days for the fitting of the bactericidal activity of isoniazid.

To validate the quality of the fitting we calculate the average coefficient of determination R^2^ for every drug. Isoniazid and ethambutol show a satisfactory R^2^ of 0.67 and 0.70 respectively and a very good 0.90 for streptomycin. The coefficient of determination for the fitting of rifampicin is rather low with 0.36. Our model overestimates the bactericidal activity of rifampicin at high concentrations and underestimates the activity at low concentrations. Apparently a single drug action model as we use it does not provide the same descriptive quality for every first line drug. No kill curves were available for pyrazinamide hence we estimate an appropriate *EC_50_* value from other studies [7,8].

**Robustness analysis**

In order to obtain a better understanding of the influences of different parameter estimates in the model we performed a robustness analysis in which we vary them and looked at their impact on the treatment outcome. In the following results we monitor the likelihood of treatment failure and the emergence of MDR-TB after a single treatment with the four standard first-line drugs. As in the main text treatment failure is again defined as incomplete sterilization after the completion of therapy and emergence of MDR-TB is defined as 10% or more [9] of the remaining population being resistant against at least isoniazid and rifampicin.

**Carrying capacity**

There is a large variance in reports about the maximum population size of *M. tuberculosis* in a human lung during acute infection. This is also based on the fact that this number depends on the host immune defense and the course of infection. Many studies report 10^9^ bacilli per open cavity and therefore an overall population size of 10^10^ or above. Thus, to study the effect of varying carrying capacity we ran simulations with compartmental carrying capacities that are 10-fold higher or lower for every compartment (see Figure 7 A and B). With 10-fold higher carrying capacities the probability for treatment failure increases substantially. The increased number of bacilli in each compartment also increases the standing variation in the bacterial population. This means that there are more bacteria that carry single or even double resistance mutations. Therefore, an increased carrying capacity also favors the emergence of MDR-TB.

**Resistance Costs**

The cost of resistance in M. tuberculosis is generally assumed to be low [10–13]. However, those estimates are mostly based on observations of clinically isolated strains. It is possible that these fitness costs are alleviated by compensatory mutations, which occur later during a chronic infection or during the chain of transmission events. Since we assumed *de novo* emergence of resistance we assigned a 10% fitness cost on reproductive success for every mutation.

We ran simulations in which we increased as well as decreased the fitness costs per resistance mutations. As we can see in Figure 7 C and D the results with slightly lower fitness costs still provide reasonable results. Although, if the costs are lower than 5% the probability of treatment failure and the likelihood of MDR emergence are unrealistically high. Thus, we conclude that resistance mutations are do very likely carry some fitness costs. Otherwise treatment outcomes would be expected to be much worse than what is generally observed in studies. This conclusion is partially confirmed in a previous study [14].

**Migration Rates**

The rate with which *M. tuberculosis* migrates among the three compartments has to our knowledge not been quantified. We follow the assumptions made by Lipsitch and Levin [15]. The migration has only a pronounced influence if it increases (see Figure 7 C and D). This is most likely due to the increased density-dependent bacterial killing in the small compartments of macrophages and granulomas. With higher migration rates these compartments are flooded with bacteria from the open cavities. However, the influence on the emergence of MDR-TB is still small. Even though our estimates for the migration rate are not well established and we consider them to be already rather high we think that its minor influence does not severely affect the validity of our model.

**EC_50_**

We also investigate the influence of the EC_50_ parameter, i.e. the efficiency of the drug. In Figure 7 G and H we vary the EC_50_ values of every drug simultaneously between 1/10, 5/10 and the five- and tenfold of the standard parameter setting. An increased EC_50_ is predicted to cause a guaranteed treatment failure even at perfect adherence. On the other hand a decreased EC_50_ may dramatically improve the likelihood of a successful treatment at adherence levels, which are far below the optimum. Figure 7 F indicates that an elevated EC_50_ only promotes the selection of MDR-TB at intermediate levels of adherence, i.e. if the drugs are able to exert a certain selective pressure.

**Missing drug**

In resource-limited settings, the drug supply is not always guaranteed, such that therapy might only comprise a subset of the four drugs. To investigate the impact of a missing drug, we test all four possible standard treatments that each lack one of the first-line drugs and assess the effect (see Figure 7 I and J). The lack of isoniazid, rifampicin or ethambutol always leads to treatment failure irrespective of the level of adherence. The explanation for this is as follows: The extracellular compartment harbors the largest number of bacteria. Here, isoniazid rifampicin and ethambutol most efficiently reduce bacterial load. Because of the high bacterial load we would expect that mutants resistant to either of these drugs pre-exist at treatment initiation. However, double-resistant mutants that could evade two drugs are expected to pre-exist only in a small fraction of drug-naive patients. If one of these main drugs is missing the mutants that are resistant against the remaining drug immediately take over and spread. Compared to isoniazid, rifampicin and ethambutol pyrazinamide is less essential for treatment success. This is probably because it does not affect the large extracellular compartment. However, it is important for clearing bacteria residing in the caseous centers of granulomas, where it is the most active drug. Therefore, its usage favors a positive treatment outcome. However, its absence does not affect the outcome as much as the other drugs.

Because the lack of isoniazid and rifampicin does not select for resistance against these drugs it is also unlikely that MDR occurs at a substantial frequency. The previously observed minor influence of pyrazinamide is also evident as its absence does not substantially increase the risk of MDR emergence. Only a regimen without ethambutol drastically increases the probability for MDR to occur. This shows how important the role of ethambutol as a third extensively effective bactericidal drug is. Ethambutol is needed to prevent widespread treatment failure due to the development of MDR-TB.

**Transmitted Resistance**

In Figure 8 we examine the possible effect of an infection with an *M. tuberculosis* strain that is already resistant at transmission. To model this, we exchange the bacterial inoculum at the beginning of the infection with a strain that is resistant to one or two drugs. It is known that patients with active TB are highly infectious and even a small inoculum of one hundred bacilli or less that comprises primarily resistant mutants might establish an infection in a susceptible host. As in every simulation the infection is simulated for one year to reach its full potential and equilibrate. During this year random reversion mutations may occur which give rise to sensitive strains. These strains may slowly outcompete resistant strains and become more frequent due to their higher fitness in absence of drugs.

The pre-existence of rifampicin or isoniazid resistance increases the risk of treatment failure the most among the single mutants at perfect adherence. This could be due to the potency of these drugs and the competitive advantage that such resistance mutations grant. Not surprisingly, a isoniazid or rifampicin resistant inoculum also increases the risk of MDR-TB. Pre-existing pyrazinamide or ethambutol resistance has almost no effect on treatment outcome. Most likely due to their minor bactericidal effectivity during the therapy. A double resistant inoculum as in Figure 8 C and D has a fatal impact. Treatment failure is almost inevitable and MDR-TB occurs especially at higher levels of adherence.

**References**

1. Gillespie DT. Exact Stochastic Simulation of Coupled Chemical Reactions. J Phys Chem. 1977;81: 2340–2361.

2. Czock D, Keller F. Mechanism-based pharmacokinetic-pharmacodynamic modeling of antimicrobial drug effects. J Pharmacokinet Pharmacodyn. 2007;34: 727–751. doi:10.1007/s10928-007-9069-x

3. de Steenwinkel JEM, de Knegt GJ, ten Kate MT, van Belkum A, Verbrugh HA, Kremer K, et al. Time-kill kinetics of anti-tuberculosis drugs, and emergence of resistance, in relation to metabolic activity of Mycobacterium tuberculosis. J Antimicrob Chemother. 2010;65: 2582–2589. doi:10.1093/jac/dkq374

4. Marcel N, Nahta A, Balganesh M. Evaluation of Killing Kinetics of Anti-Tuberculosis Drugs on Mycobacterium tuberculosis Using a Bacteriophage-Based Assay. Chemotherapy. 2008;54: 404–411. doi:10.1159/000153314

5. Dhillon J, Mitchison DA. Activity and Penetration of Antituberculosis Drugs in Mouse Peritoneal Macrophages Infected with Mycobacterium microti OV254. Antimicrob Agents Chemother. 1989;33: 1255–1259. Available: http://www.pubmedcentral.nih.gov/articlerender.fcgi?artid=172636&tool=pmcentrez&rendertype=abstract

6. Rastogi N, Labrousse V, Goh KS. In Vitro Activities of Fourteen Antimicrobial Agents Against Drug Susceptible and Resistant Clinical Isolates of Mycobacterium tuberculosis and Comparative Intracellular Activities Against the Virulent H37Rv Strain in Human Macrophages. Curr Microbiol. 1996;33: 167–175. Available: http://www.ncbi.nlm.nih.gov/pubmed/8672093

7. Zhang Y, Mitchison DA. The curious characteristics of pyrazinamide: a review. Int J Tuberc Lung Dis. 2003;7: 6–21. Available: http://www.ncbi.nlm.nih.gov/pubmed/12701830

8. Heifets LB. Antituberculosis Drugs: Antimicrobial Activity In Vitro. In: Heifets LB, editor. Drug Susceptibility in the Chemotherapy of Mycobacterial Infections. 1st ed. Boca Raton: CRC Press; 1991. pp. 13–58.

9. Kim SJ. Drug-susceptibility testing in tuberculosis: methods and reliability of results. Eur Respir J. 2005;25: 564–569. doi:10.1183/09031936.05.00111304

10. Pym AS, Saint-Joanis B, Cole ST. Effect of katG Mutations on the Virulence of Mycobacterium tuberculosis and the Implication for Transmission in Humans. Infect Immun. 2002;70: 4955–4960. doi:10.1128/IAI.70.9.4955

11. Cohen T, Sommers B, Murray MB. The effect of drug resistance on the fitness of Mycobacterium tuberculosis. Lancet - Infect Dis. 2003;3: 13–21. Available: http://www.ncbi.nlm.nih.gov/pubmed/12505028

12. Andersson DI. The biological cost of mutational antibiotic resistance: any practical conclusions? Curr Opin Microbiol. 2006;9: 461–465. doi:10.1016/j.mib.2006.07.002

13. Gagneux S, Long CD, Small PM, Van T, Schoolnik GK, Bohannan BJM. The Competitive Cost of Antibiotic Resistance in Mycobacterium tuberculosis. Science (80- ). 2006;312: 1944–1946. doi:10.1126/science.1124410

14. Davies AP, Billington OJ, Bannister BA, Weir WR, McHugh TD, Gillespie SH. Comparison of Fitness of Two Isolates of Mycobacterium tuberculosis, one of Which had Developed Multi-drug Resistance During the Course of Treatment. Br Infect Soc. 2000;41: 184–187. doi:10.1053/jinf.2000.0711

15. Lipsitch M, Levin BR. Population dynamics of tuberculosis treatment: mathematical models of the roles of non-compliance and bacterial heterogeneity in the evolution of drug resistance. Int J Tuberc Lung Dis. 1998;2: 187–199. Available: http://www.ncbi.nlm.nih.gov/pubmed/9526190

**Figure 6. Comparison of the concentration- and time-dependent effects of isoniazid, rifampicin, ethambutol and streptomycin on sensitive *M. tuberculosis*.** The plots in the left column are experimentally obtained killing curves for anti-tuberculosis drugs by de Steenwinkel *et al.* [3] and Marcel *et al.* [4] and originate from a metabolically highly active strain of Mtb H37Rv cultured *in vitro* at 37°C [3]. The plots on the right show the simulated killing curves that were calculated by fitting the pharmacodynamic model to the experimental data. The fitting was done by minimizing the sum of least squares over all curves. The coefficient of determination (R^2^) indicates the average goodness of fit for each drug. Greyed out lines were not used for fitting. Modified from [3].

**Figure 7. Sensitivity analysis of the probability of treatment failure and emergence of resistance.** In the left column are the plots for the probability of treatment failure due to incomplete clearance and in the right column are the plots for the probability of the emergence of an MDR-TB strain, which accounts for at least 10% of the overall population. (A and B) Effect of different carrying capacities. (C and D) Effect of different fitness costs per resistance mutation. (E and F) Effect of different migration rates among compartments. (G and H) Effect of lower or higher EC_50_ values. (I and J) Effect of treatment consisting of only three drugs.

**Figure 8. Influence of pre-existing resistance mutations on treatment outcome after a regular six-month therapy.** In the left column are the plots for the probability of treatment failure due to incomplete clearance and in the right column are the plots for the probability of the emergence of an MDR-TB strain, which accounts for at least 10% of the overall population. (A and B) Effect of a homogeneous inoculum consisting of genotypes resistant to one drug. (C and D) Effect of a homogeneous inoculum consisting of genotypes resistant to two drugs.
